# Diagnostic performance and interreader agreement of individual and combined non-enhanced and contrast-enhanced MR imaging parameters in adhesive capsulitis of the shoulder

**DOI:** 10.1007/s00256-023-04391-8

**Published:** 2023-07-03

**Authors:** Bernd Erber, Nina Hesse, Sophia Goller, Fabian Gilbert, Jens Ricke, Christian Glaser, Andreas Heuck

**Affiliations:** 1 Department of Radiology, University Hospital, LMU Munich, Marchioninistr. 15, 81377 Munich, Germany; 2 Department of Orthopaedics and Trauma Surgery, Musculoskeletal University Center Munich (MUM), University Hospital, LMU Munich, 81377 Munich, Germany; 3 Radiologisches Zentrum München, Pippinger Str. 25, 81245 Munich, Germany

**Keywords:** MR imaging, Adhesive capsulitis of the shoulder, Diagnostic performance, Interreader agreement, Contrast enhancement

## Abstract

**Objective:**

The aims of our study were to analyze agreement among readers with different levels of expertise and diagnostic performance of individual and combined imaging signs for the diagnosis of adhesive capsulitis of the shoulder.

**Methods:**

In a retrospective study, contrast-enhanced shoulder MRIs of 60 patients with and 120 without clinically diagnosed adhesive capsulitis were evaluated by three readers independently. As non-enhanced imaging signs, readers evaluated signal intensity and thickness of the axillary recess capsule, thickness of the rotator interval capsule and the coracohumeral ligament as well as obliteration of subcoracoid fat. Furthermore, contrast enhancement of axillary recess and rotator interval capsule were evaluated. Data analysis included interreader reliability, ROC analysis, and logistic regression (*p* < 0.05).

**Results:**

Contrast-enhanced parameters showed substantially higher agreement among readers (ICC 0.79–0.80) than non-enhanced parameters (0.37–0.45). AUCs of contrast-enhanced signs (95.1–96.6%) were significantly higher (*p* < 0.01) than of non-enhanced imaging signs (61.5–85.9%) when considered individually. Combined evaluation of axillary recess signal intensity and thicknesses of axillary recess or rotator interval—when at least one of two signs was rated positive—increased accuracy compared to individual imaging signs, however not statistically significant.

**Conclusion:**

Contrast-enhanced imaging signs show both distinctly higher agreement among readers and distinctly higher diagnostic performance compared to non-enhanced imaging signs based on the imaging protocol used in this study. Combined evaluation of parameters showed a tendency to increase discrimination; however, the effect on diagnosis of ACS was not statistically significant.

## Introduction

Adhesive capsulitis of the shoulder (ACS) is a common disease in the middle-aged population where patients suffer from painfully reduced active and passive shoulder range of motion [1–5]. Currently, conservative management with physiotherapy and nonsteroidal anti-inflammatory drugs is preferred over arthrolysis or embolization, reserved for cases refractory to conservative treatment [6, 7]. Thus, timely initiation of conservative therapy is fundamental to obviate a protracted course or invasive treatment possibly associated with an unfavorable outcome in this normally self-limiting disease. [1] Therefore, proper clinical and radiological diagnosis of ACS is important [4, 8, 9] and has an impact on patient management [10].

However, there is still controversy about the diagnostic performance of the individual MR imaging signs of capsulitis including the potential benefit of IV contrast application, illustrated by the results of some recent studies [1, 5, 11–13]. Chi et al. [13] claimed high performance of non-enhanced imaging, and Ahn et al. [11] found similar performance of non-enhanced and CE imaging, respectively. On the other hand, it was shown that the accuracy of imaging signs in contrast-enhanced (CE) MR sequences was superior to imaging signs in non-enhanced sequences [5, 11, 12]; e.g., Pessis et al. [12] found significantly higher area under the curve (AUC) values for CE of the joint capsule in the axillary recess (0.986) and rotator interval (0.921) than for T2 hyperintensity in both locations (0.927 and 0.7). Except a basic analysis in the study of Chi et al. [13], imaging signs were evaluated individually. However, when different signs in the same patient are contradictory regarding the presence of ACS, this may cause inconsistent interpretation by radiologists in view of the clinically relevant decision whether patients suffer from ACS or not.

Also, diagnostic agreement among readers is becoming more and more recognized—and requested—as relevant for the value of radiological imaging [14] in view of a uniform and stratified patient management and therapy. This may be all the more relevant in day-to-day clinical practice when different levels of experience are to be considered.

Therefore, the first aim of our study was to analyze the diagnostic performance and agreement across readers with different levels of expertise for established signs of ACS in non-enhanced and CE MR imaging.

Our second aim was to investigate whether the diagnostic performance for ACS can be improved by pairwise combined evaluation of individual MR imaging signs (with at least one of both being positive for correct diagnosis of ACS) and to test whether these combinations of non-enhanced MRI parameters can live up to combined CE MRI.

## Methods

### Patients

The study group consisted of a total of 180 patients who were referred to our outpatient radiology institution and who all received non-enhanced and CE MRI of the shoulder on request of their referring physicians between January 2019 and December 2020. Sixty patients of this group were clinically diagnosed for ACS by experienced orthopedic surgeons based on the consensus definition of Zuckerman et al. [4] which served as the standard of reference, and as done before [5]: shoulder pain (1), limited active and passive shoulder range of motion (ROM) consisting of reduced anterior flexion (2) and abduction (3) (less than 90° anterior flexion and abduction), and reduced external (4) and internal (5) rotation (less than 50% ER and IR of the contralateral shoulder). The standard of reference was considered positive if 4 of 5 of these signs were clinically present.

The control group consisted of 120 randomly chosen patients without clinical signs (no limited active and passive shoulder range of motion) of ACS who were referred to our outpatient institution for non-enhanced and CE MRI of the shoulder in the same time period for various other indications. Patients with osteoarthritis > grade 1 according to the Kellgren-Lawrence classification, malignant pre-existing disease, and clear signs of synovitis were excluded as these conditions might clinically overlap with ACS [1]. All 180 patients had MRI on the same scanners and with the same imaging protocol. Patients with an age under 18 years were not included. Approval from the institutional review board was obtained. Because of the retrospective character of the study and complete anonymization of all patient-related data, no patient consent to the study was requested by the board.

### MR imaging protocol

MR imaging of all shoulder joints was performed on two 3 T scanners (both: Magnetom Skyra; Siemens Erlangen, Germany) using a dedicated 15-channel shoulder coil. The imaging protocol consisted of the following sequences: non-CE oblique coronal T1-weighted (T1w) fast spin echo (TR/TE of 650–700/10 ms; ETL (echo train length) 4), fat saturated oblique coronal and axial proton density weighted (PDw fat sat) fast spin echo (TR/TE of 3800–4000/36 ms; ETL 9), a T2w oblique sagittal (TR/TE 5000/103 ms; ETL 14) fast spin echo, and a T1w fat sat oblique sagittal fast spin echo sequences immediately after IV contrast administration (ETL 4). For all sequences, a field of view (FOV) of 16 cm and a slice thickness of 3 mm was used.

### Image review and analysis

Image review and quantitative analysis were performed using a commercially available picture archiving and communication system (Jivex; Visus Health IT, Bochum, Germany). All MR studies were anonymized and independently reviewed by three radiologists blinded to all clinical information and with different experience in musculoskeletal imaging. To avoid interpretation bias, ACS cases and control cases were pooled together and the order of reading of the total of 180 studies was randomly assigned.

Reader 1 was a resident with 3 years of experience in radiology, reader 2 a board-certified radiologist with 8 years of experience. Reader 3 was an expert consultant in musculoskeletal imaging with more than 20 years of experience. To avoid bias in evaluating non-enhanced images together with information from CE sequences, image analysis was split up in two sessions. First, only non-enhanced sequences were evaluated, and in a second session 5 weeks later, CE sequences were interpreted. All evaluated MR imaging features had been shown to be relevant for the diagnosis of ACS in the current literature. For non-enhanced sequences, the following features were used:

1. Increased signal intensity of the axillary recess capsule on coronal PDw fat sat sequences (0 = no signal alteration; 1 = increased signal intensity compared to surrounding muscle) [8, 9, 13, 15–17].
2. Greatest thickness of the joint capsule of the axillary recess as determined in coronal PDw fat sat sequences.
3. Greatest thickness of the joint capsule in the rotator interval (RI thickness) as determined in sagittal T2w sequences [8, 9, 11, 16].
4. Greatest thickness of the coracohumeral ligament (CHL thickness) as determined in sagittal T2w sequences [1, 13, 15, 17–19] and
5. Obliteration of the subcoracoid fat triangle (SCF obliteration) as assessed in sagittal T1w and T2w sequences (0 = no obliteration; 1 = mild obliteration with < 25% replacement of fat signal; 2 = moderate obliteration with 25–50% replacement of fat signal; 3 = severe obliteration with > 50% replacement of fat signal) [1, 13, 18, 20].

**Fig. 1:**
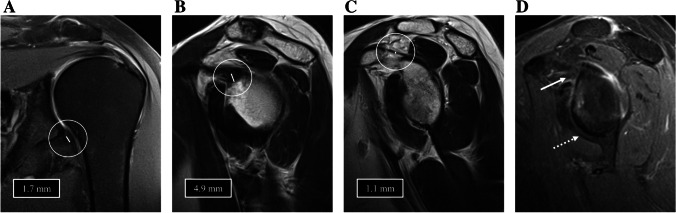
Images of the left shoulder of a 45-year-old female without clinical and MR morphological signs of ACS. **A** Coronal PD-weighted image showing a regular capsule at the axillary recess without thickening (1.7 mm) or hyperintensity. **B** Sagittal T2-weighted image shows a slightly thickened capsule at the rotatory interval (4.9 mm) and **C** a regularly thin CHL (1.1 mm) as well as non-obliterated subcoracoid fat. **D** T1-weighted fat saturated contrast-enhanced sagittal image is negative for contrast enhancement in the axillary recess (dotted arrow) and rotatory interval (arrow)

**Fig. 2:**
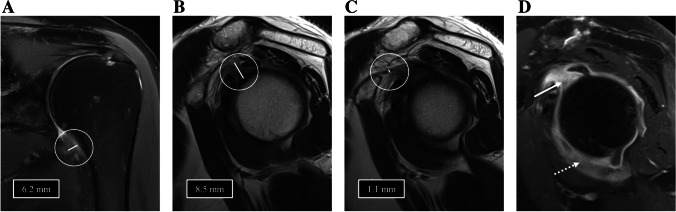
Images of the left shoulder of a 49-year-old male with clinical signs of ACS. **A** Coronal PD-weighted image shows a thickened capsule at the axillary recess (6.2 mm) with increased signal intensity while **B** T2-weighted image shows an increased thickness of the capsule at the rotatory interval (8.5 mm) indicating the presence of ACS. In contrast, the CHL is not thickened (1.1 mm), and the subcoracoid fat is not obliterated in T2-weighted sagittal imaging (**C**). **D** In T1-weighted fat-saturated contrast-enhanced sagittal image, axillary recess (dotted arrow) and rotatory interval (arrow) present with distinct contrast uptake confirming the diagnosis of ACS

**Fig. 3:**
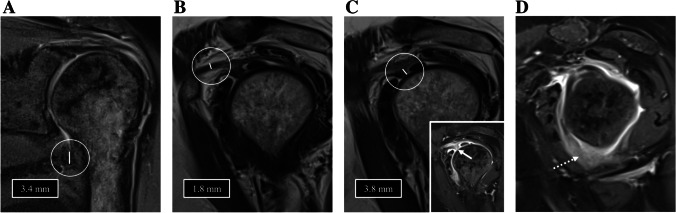
Images of the right shoulder of a 58-year-old female with clinical signs of ACS. **A** Coronal PD-weighted image shows a non-thickened capsule at the axillary recess with predominantly regular signal intensity. The CHL (**B**) (1.8 mm) as well as the capsule at the rotatory interval (**C**) are not thickened in T2-weighted sagittal imaging. However, the rotatory interval (**C**, arrow in small image on bottom right) and the axillary recess (**D**, dotted arrow) show distinct contrast uptake in T1-weighted fat-saturated contrast-enhanced sagittal imaging indicating the presence of ACS

Since—to the best of our knowledge—no standardized definitions exist in which location exactly thickness measurements should be taken, readers were instructed to take them at points where they felt that the respective structure showed its greatest transverse extension. In ACS, both the capsule and the adjacent synovium are inflamed and cannot be clearly differentiated from each other on MR images [1]. However, also under physiological conditions, both structures cannot be distinguished with MRI. Therefore, capsule and synovium were measured together in cases with and without ACS. For CE sequences, readers evaluated these features:

Contrast enhancement in (A) axillary recess (axillary recess CE) and (B) in RI (RI CE) as determined in contrast-enhanced sagittal T1w fat sat sequences. Contrast enhancement was considered present when the joint capsule displayed higher signal than the adjacent muscle [12, 21]. When present, contrast enhancement was graded as follows: grade 1 = mild enhancement; grade 2 = moderate enhancement; grade 3 = strong enhancement) [1, 8, 11, 16, 18]. In Figs. 1, 2, and 3, examples of patients without (Fig. 1) and with (Figs. 2 and 3) clinical and MR morphological signs of ACS are displayed.

### Pairwise combination of imaging signs

To investigate whether the diagnostic performance of individually assessed imaging signs can be improved by their pairwise combined evaluation, the three best-performing combinations of imaging signs were analyzed: axillary recess signal intensity and RI thickness; axillary recess signal intensity and axillary recess thickness; axillary recess thickness and RI thickness. Thereby, rating either one or both of the combined imaging signs as positive was considered as a correct diagnosis of ACS. Conversely, both had to be negative for the correct diagnosis of no ACS.

### Statistical analysis

Statistical analysis was performed using R-Studio (Version 4.0.4, RStudio Inc., Boston, MA). 

As surrogate parameter for agreement among readers, the interreader reliability using ICC and kappa statistics, respectively, was determined. For the evaluated raw values (axillary recess thickness, RI thickness, and CHL thickness in millimeter (mm); axillary recess signal intensity, SCF obliteration, and axillary recess CE and RI CE), the ICC was calculated.

For better comparability of the different (ordinary and metric) scale levels of the various imaging signs, all raw values were transferred in binary values depending on the calculated optimal cutoffs of our readers (0 = below cutoff values/no signal alteration or enhancement; 1 = above cutoff values/increased signal or enhancement): for axillary recess thickness, a cutoff of ≥ 4 mm was used; for RI thickness, cutoff values of ≥ 5 mm and ≥ 6 mm were used because both showed the highest interreader reliabilities as further discussed in “Results” and “Discussion” sections. For CHL thickness, a cutoff of ≥ 3 mm was used.

The degree of agreement was classified using kappa values according to Landis and Koch [22] and transferred to ICC as follows: 0.21–0.40 fair agreement; 0.41–0.60 moderate agreement; 0.61–0.80, substantial agreement; and 0.81–1.00, almost-perfect agreement.

Student’s *t* test for independent samples was applied to test for significant differences of all parameters between ACS and control groups.

Receiver operating characteristic (ROC) analyses were used to evaluate the diagnostic performance of each individual sign for ACS by calculating the area under the curve (AUC). 95% confidence intervals and method of DeLong [23] and Sun et al. [24] (R-package roc.test) were used to test for statistical significance. Based on ROC analyses, optimal cutoffs were calculated for all imaging signs and all readers. Sensitivities, specificities, and true positive and true negative rates for individual imaging signs were calculated.

The diagnostic performance of pairwise combined non-enhanced and CE imaging signs was calculated using ROC analyses based on logistic regression. For the three best-performing combinations of non-enhanced imaging signs (axillary recess signal intensity and RI thickness; axillary recess signal intensity and axillary recess thickness; axillary recess thickness and RI thickness) and for the combination of axillary recess CE and RI CE sensitivities, specificities, and true positive (TPR) and true negative rates (TNR) were calculated. The presence of both or at least one positive imaging sign was rated as indicative for ACS.

A multivariate logistic regression model was implemented to evaluate the potential statistically significant contribution of the imaging signs to the model (diagnosis of ACS). A *p* value of 0.05 was set as the limit of statistical significance.

## Results

### Patients

Out of the 180 patients, 60 (29 female and 31 male) had signs of ACS as defined by the standard of reference; all patients with ACS suffered from significantly limited active and passive ROM and all but two presented with pain in clinical examination. This combination of symptoms is predominantly associated with active stages 1–3 [1]. One hundred twenty (61 female and 59 male) had no signs of ACS. Mean patient age in the ACS group was 59 (SD ± 10.8) years and 58 (SD ± 12.1) years in the control group.

### Agreement among readers

All ICC and kappa values are shown in Table 1.

**Table 1:** Reader agreement. Columns showing intraclass correlation (ICC) and Fleiss kappa (kappa) values of all 3 readers for each imaging sign of ACS (rows)

	ICC	ICC 95% CI	Kappa	Kappa 95% CI
AR SI:	0.42	0.37–0.46	0.42	0.33–0.50
AR thickness:	0.45	0.41–0.49	0.36	0.27–0.45
RI thickness:	0.37	0.32–0.41	0.22	0.14–0.31
CHL thickness:	0.41	0.37–0.45	0.26	0.16–0.33
SCF obliteration:	0.49	0.45–0.53	0.39	0.30–0.47
AR CE	0.80	0.78–0.82	0.79	0.70–0.87
RI CE	0.79	0.77–0.81	0.64	0.44–0.85
AR SI + AR thickness	n.a	n.a	0.33	0.25–0.42
AR SI + RI thickness	n.a	n.a	0.47	0.39–0.56
AR CE + RI CE	n.a	n.a	0.77	0.69–0.86

*AR* axillary recess, *SI* signal intensity, *RI* rotator interval, *CHL* coracohumeral ligament, *SCF* subcoracoid fat, *CE* contrast enhancement

Using 95% confidence intervals, interobserver reliability calculated with ICC was significantly higher for CE of axillary recess (0,80; 95% CI 0.78–0.82) or CE of RI (0.79; 95% CI 0.77–0.81) than for all non-enhanced imaging signs (0.37–0.49).

Interobserver reliability using kappa statistics for binary values was higher for both CE imaging sings compared to all non-enhanced imaging signs (0.22–0.42), however, using 95% confidence interval only significantly for CE of axillary recess (0.79; 95% CI 0.70–0.87).

Interobserver reliability for the combination of axillary recess signal intensity and RI thickness (0.47; 95% CI 0.39–0.56) using kappa statistics was slightly higher than for all individual parameters, however with overlapping 95% CI.

In summary, ICC/kappa of enhancement of axillary recess and RI showed at least substantial agreement while ICC/kappa of non-enhanced imaging signs ranged between fair and moderate.

### Diagnostic performance of individual imaging signs

Sensitivities, specificities, TPR, and TNR for evaluation of all individual and pairwise combined imaging signs for all readers are listed in Table 2.

**Table 2:** Diagnostic performance. Columns showing AUC (area under the curve), sensitivity, specificity, TPR (true positive rate), and TNR (true negative rate) for individual imaging signs and best combinations of imaging signs of ACS (rows). In each box, values of readers 1, 2, and 3 (from left to right) are shown

	AUC			Sensitivity			Specificity			TPR			TNR		
	Reader 1	Reader 2	Reader 3	Reader 1	Reader 2	Reader 3	Reader 1	Reader 2	Reader 3	Reader 1	Reader 2	Reader 3	Reader 1	Reader 2	Reader 3
AR signal intensity:	73.3	79.9	85.9	53.3	75.0	78.3	93.1	78.8	94.1	80.0	64.3	87.0	80.0	86.4	89.7
AR thickness:	71.9	69.6	81.7	60.0	71.7	76.3	69.5	54.2	71.3	50.9	43.9	57.5	77.8	79.3	86.0
RI thickness:	67.9	62.0	76.5	66.7	65.0	65.0	61.0	55.1	74.6	46.0	42.0	55.7	78.5	75.9	80.9
CHL thickness:	63.0	54.0	61.5	73.3	48.3	78.0	49.2	61.9	40.7	41.9	45.7	39.8	78.7	70.9	79.0
SCF obliteration	64.1	56.0	56.6	73.3	58.3	66.6	45.0	53.3	41.7	40.0	38.5	32.4	77.1	71.9	66.0
AR contrast enhancement	90.3	91.6	96.6	85.0	88.3	96.7	91.5	88.0	92.2	83.6	79.1	86.6	92.4	93.8	98.2
RI contrast enhancement	91.1	91.2	95.1	88.3	90.0	91.7	85.5	81.6	96.6	91.5	71.1	93.2	87.2	81.7	95.9
AR SI + RI thickness	81.3	83.9	92.3	71.7	80.0	81.7	80.8	75.8	89.2	66.1	62.3	79.0	85.2	88.4	90.7
AR SI + AR thickness	79.3	82.0	89.8	63.3	75.0	80.0	87.5	67.5	90.0	71.7	53.6	80.0	82.7	84.4	90.0
AR + RI contrast enhancement:	94.9	96.4	98.2	88.3	93.3	98.3	90.8	85.0	90.8	82.8	75.7	84.3	94.0	96.2	99.1

*AR* axillary recess, *SI* signal intensity, *RI* rotator interval, *CHL* coracohumeral ligament, *SCF* subcoracoid fat, *CE* contrast enhancement

Using increased signal intensity of the axillary capsule as indicator for ACS a sensitivity of 78.3% and a specificity of 94.1% was found for the expert. Corresponding calculations for the two less experienced readers for all imaging signs were consistently slightly below the values of the expert.

For axillary recess capsular thickness, an optimal cutoff of ≥ 4 mm as indicative for ACS (sensitivity 76.3%; specificity 71.3%) was found. This was also the optimal cutoff for both less experienced readers.

For RI thickness, an optimal cutoff of ≥ 5 mm as indicative for ACS (sensitivity 65.0%; specificity 74.6%) was calculated. However, reader 1 (resident) had an optimal cutoff of ≥ 6 mm and reader 2 (board-certified radiologist) of ≥ 4 mm for RI thickness.

For CHL thickness, an optimal cutoff of ≥ 3 mm as indicative for ACS (sensitivity 78.0%; specificity 40.7%) was calculated for the expert. This was also the optimal cutoff for both less experienced readers.

For SCF obliteration, using all grades of obliteration (mild, moderate, and severe obliteration) as indicator for ACS showed highest sensitivity of 66.6% and specificity of 41.7% for the expert.

Using any contrast enhancement (mild, moderate, or strong) as indicator for ACS, a sensitivity of 96.7% and specificity of 92.2 were calculated for the expert consultant when the axillary recess was evaluated; when the rotator interval was evaluated, the sensitivity was 91.7% and specificity 96.6%, respectively.

Differences between measurements of patients with ACS and without ACS were statistically significant for all imaging signs except SCF obliteration for readers 1 and 3 and CHL thickness for reader 2 (*p* values: axillary recess SI *p* < 0.001; axillary recess thickness *p* < 0.001; RI thickness *p* < 0.01–*p* < 0.001; CHL thickness (readers 1 and 3) *p* < 0.01, CHL thickness (reader 2) *p* = 0.30; obliteration of SCF (readers 2 and 3): *p* > 0.11, obliteration of SCF (reader 1): *p* = 0.01; axillary recess CE < 0.001; RI CE *p* < 0.001).

Diagnostic performances of both CE imaging signs were significantly higher than of all individual non-CE imaging signs (reader 1: *p* < 0.001; reader 2: *p* < 0.01; reader 3: *p* < 0.001). No statistically significant difference was found between individual non-enhanced imaging signs throughout all readers.

**Fig. 4:**
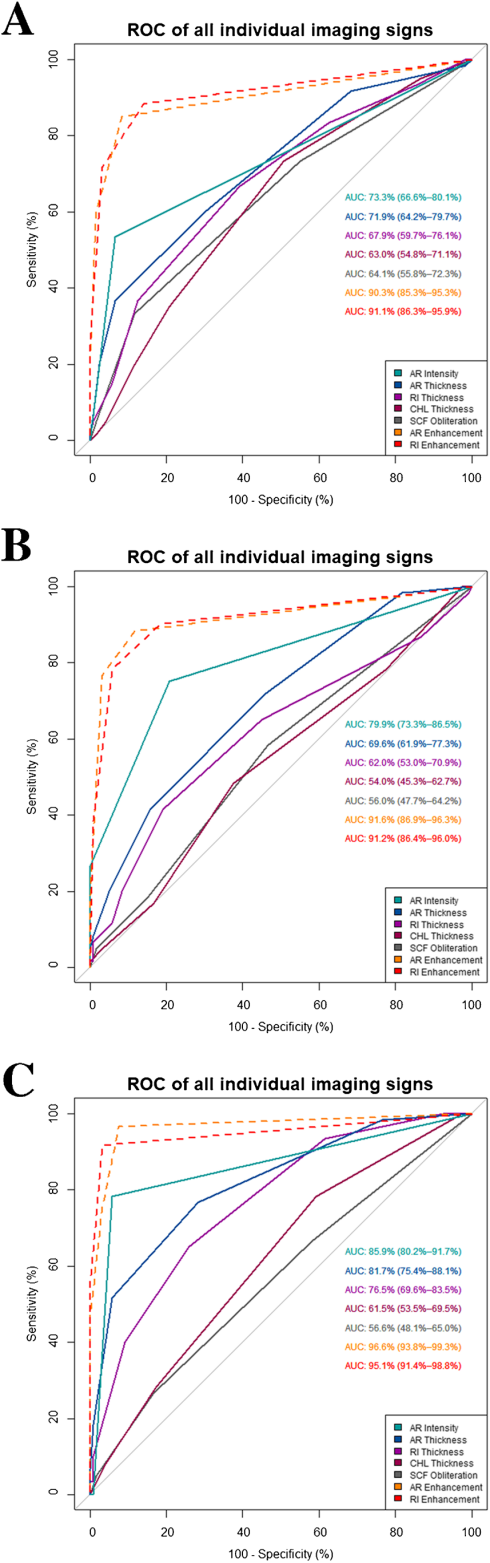
ROCs for diagnostic performance of individual imaging signs. AUCs with confidence intervals (in brackets) for all individual imaging signs for a resident (**A**; reader 1), board-certified radiologist (**B**; reader 2), and expert consultant (**C**; reader 3). AR, axillary recess; RI, rotator interval; SCF, subcoracoid fat; CHL, coracohumeral ligament

ROC curves including AUC values and 95% confidence intervals for individual evaluation of imaging signs for all readers are displayed in Fig. 4. Using 95% confidence intervals of ROC analysis for statistical comparison, no statistically relevant difference in interpretation of imaging signs of ACS was found between readers.

A multivariate logistic regression model for the combination of all ACS parameters showed that axillary recess thickness, axillary recess CE, and RI CE contributed significantly to the model (*p* values 0.034; 0.037; < 0.001). Regression analysis revealed a high diagnostic accuracy to evaluate the presence or absence of ACS using all measured parameters (*R*^2^ = 0.92) as well as both CE parameters (*R*^2^ = 0.89) which were significantly higher than that of non-enhanced parameters alone (*R*^2^ = 0.63) according to the likelihood ratio test (*p* < 0.05).

In 13 of our MR studies of patients with ACS in which axillary recess signal intensity (13/60; 21.7%) was rated negative/normal, axillary recess CE was rated positive in 84.6% (11/13) and RI CE was rated positive in 100% (13/13) by the musculoskeletal expert. In 14 MRIs with ACS in which axillary recess thickness was measured below the optimal cutoff (14/60; 23.3%), axillary recess CE was rated positive in 92.9% (13/14) and RI CE in 78.6% (11/14). In 21 MRIs with ACS in which RI thickness was measured below the optimal cutoff (21/60; 35.0%), axillary recess CE was rated correctly positive in 100% (21/21) and RI CE in 85.7% (18/21).

In shoulders with positive findings for axillary signal intensity but not for thickness of the rotator interval, 18/60 had ACS while 5/120 had no ACS. In shoulders with positive findings for thickness of the rotator interval but not for axillary signal intensity, 10/60 had ACS while 28/120 had no ACS for the expert reader.

### Diagnostic performance of combined evaluation of imaging signs

Combined evaluation of increased axillary recess signal intensity and increased RI thickness (≥ 7 mm) resulted in highest accuracy of all combined non-CE imaging parameters (a sensitivity of 81.7 and specificity of 89.2%).

For combination with axillary recess signal intensity, a cutoff ≥ 5 mm for axillary recess thickness showed highest accuracy (sensitivity of 80.0% and specificity of 90.0%).

For combined evaluation of axillary recess thickness and RI thickness, the highest performance was calculated for two combinations of cutoffs: axillary recess thickness ≥ 5 mm and RI thickness ≥ 6 mm lead to a sensitivity of 70.0% and specificity of 85.8%. Axillary recess thickness ≥ 4 mm and RI thickness ≥ 7 mm lead to a sensitivity of 80.0% and specificity of 68.3% for the expert.

**Fig. 5:**
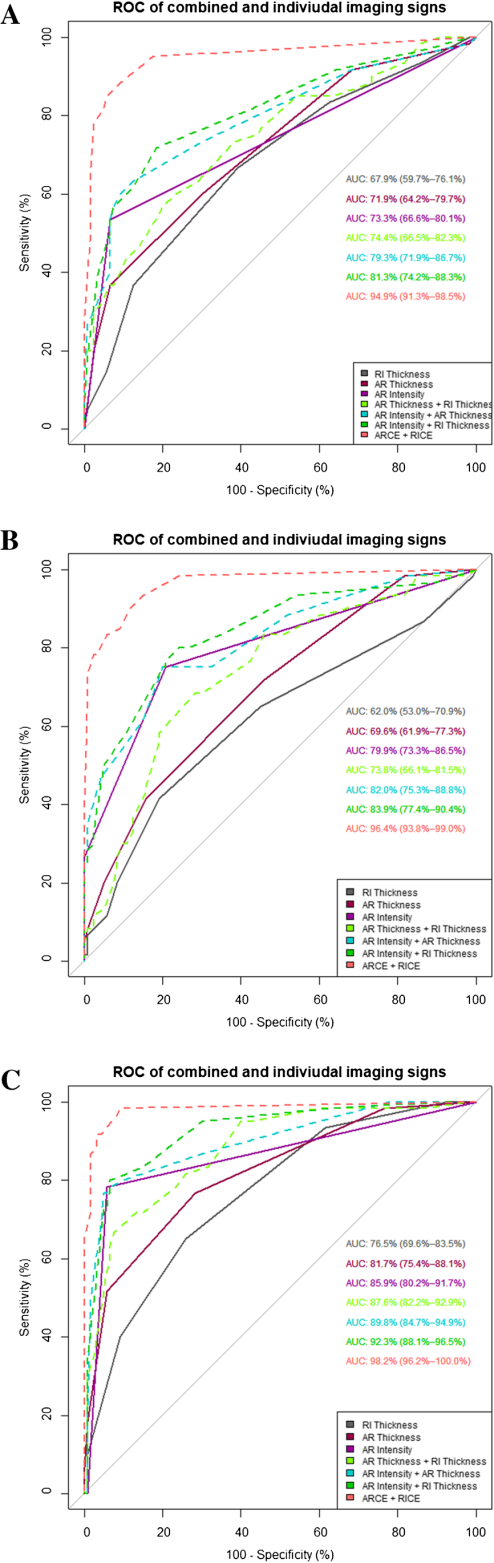
ROCs for diagnostic performance compared between individual and pairwise combined imaging signs. AUCs with confidence intervals (in brackets) for individual non-enhanced imaging signs with highest accuracy and pairwise combinations of these three signs and combination of AR and RI contrast enhancement (AR and RI CE) for a resident (**A**; reader 1), board-certified radiologist (**B**; reader 2), and expert consultant (C; reader 3). AR, axillary recess; RI, rotator interval

ROC curves including AUC values and 95% confidence intervals for the diagnostic performance of combined evaluation of imaging signs are presented in Fig. 5.

Combined evaluation of axillary recess CE and RI CE leads to the highest diagnostic performance among all combined parameters (AUC 98.2%).

Diagnostic performance of both combined CE imaging signs was significantly higher than of the abovementioned three best-performing combinations of individual imaging signs (reader 1: *p* < 0.001; reader 2: *p* < 0.001; reader 3: *p* < 0.001). No statistically significant difference was found between the three best-performing combinations of non-enhanced imaging signs and best-performing individual non-enhanced imaging signs for all readers.

Similar to the individual imaging signs, performances for pairwise combined imaging signs derived from the two less experienced readers were slightly lower than for the expert consultant; however, for all readers, the diagnostic performance of imaging signs showed the same gradation. Using 95% confidence intervals of ROC analysis for statistical comparison, no statistically relevant difference in interpretation of combined imaging signs of ACS was found between readers.

When considering all non-enhanced imaging signs together and rating the presence of at least one positive sign (above mentioned cutoffs) as indicative for ACS, sensitivity increases to 98.3% while specificity decreases to 29.2% (TPR 40.9%; TNR 92.3%). On the other hand, when considering all non-enhanced imaging signs together and solely rating the presence of all signs positive (above mentioned cutoffs) as indicative for ACS and at least rating one sign negative as indicative for no presence of ACS, sensitivity decreases to 16.7% and specificity increases to 100% (TPR 100%, TNR 0%).

## Discussion

Although ACS is considered primarily a clinical diagnosis, MRI is frequently requested to confirm the suspicion and thereby exclude other pathologies of the shoulder which might cause similar symptoms [1, 4, 20]. Moreover, MRI is often requested to identify adhesive capsulitis as a coexistent condition with another clinically suspected lesion, where the presence of adhesive capsulitis affects the management of the other pathology. This becomes even more relevant as ACS usually is self-limiting, however often with a prolonged clinical course and a resistance to therapy, or sometimes too aggressive conservative treatment which may lead to an unfavorable outcome [1, 19]. As a correct and reliable radiological diagnosis is fundamental, MRI-based diagnosis is still in the focus of research [1, 5, 11, 12]. In this study, we aimed to analyze the agreement among readers with different levels of expertise when evaluating established signs of ACS in non-enhanced and CE MR imaging studies. In addition to analyzing the diagnostic performance of these individual signs with and without CE in the context of the current literature, we wanted to investigate whether the performance of both non-enhanced and CE imaging can be increased by pairwise combining individual imaging signs of ACS to establish the diagnosis.

In our study, agreement between readers was found to be substantial or better only for the two CE parameters. This is partially in line with findings of previous studies. For example, Ahn et al. [11] found similar kappa values for evaluation of axillary recess CE and RI CE (*k* = 0.92 and *k* = 0.81) compared to our study (*k* = 0.79 and *k* = 0.64; ICC 0.80 and 0.79), but they found a similarly high correlation for axillary recess thickness (Pearson’s coefficient *r* = 0.90) and axillary recess signal intensity (*k* = 80). This is in contrast to our findings for both ICC (0.45 and 0.42) and kappa statistics (*k* = 0.36 and *k* = 0.42) indicating only fair-to-moderate agreement. Pessis et al. [12], too, found almost perfect and substantial agreement for non-enhanced parameters (kappa = 0.94 for axillary recess signal intensity and ICC 0.85 for axillary recess thickness; ICC of RI thickness of 0.6). This is an interesting result, as we clearly defined measurement of imaging signs prior to reader evaluation which should increase agreement among readers. One explanation could be that in our study, evaluation was performed by three independent readers. The difference to Pessis et al. who had a junior radiologist (1-year experience) and a senior radiologist (21 years of experience) as readers and Ahn et al. [11] (5 and 11 years of experience) could be explained by the fact that generally, robust estimation of interrater agreement is dependent on the number of readers [25].

Furthermore, we transferred the raw values of all imaging signs into binary values depending on whether they exceeded cutoffs of the current literature or not as described in “Methods” section. This is clinically relevant as radiologists in their day-to-day practice have to make the decision whether a shoulder MRI is positive or negative for signs of ACS; all the more on the background of interrater agreement in view of different levels of expertise of radiologists.

As described in “Methods” section, imaging signs differ in their scale levels (categorical vs. continuous). While perceived axillary recess signal intensity is a categorical variable with only two characteristics and therefore has a clear cutoff, cutoffs for quantitative parameters such as measured RI thickness are more difficult to use in routine imaging because they show a broader variability, both in the current literature [1, 18] and in this study. Results for RI thickness showed substantial variability (cutoffs from 4 to 6 mm) between the three readers in our study, and these cutoffs furthermore differed from the literature which is reflected by the values proposed by Mengiardi et al. with an optimal cutoff of ≥ 7 mm [26] and Pessis et al. with a cutoff > 3 mm [12]. This variance was confirmed by a poor interobserver reliability in our study of only 0.22 for the diagnosis of ACS using RI thickness. Obviously, the individual perception of what is a thickened rotatory interval varies substantially between radiologists. One reason could be the more complex anatomy of the rotator interval compared to the axillary recess. This, however, does not preclude that the individual radiologists reach a similar conclusion and diagnosis starting out from their individual perception. It rather underlines how difficult it is to make decisions based on cutoffs from the literature. This also shows the advantage of categorical/binary parameters. In this view, our results bring together the substantial differences between cutoff values reported in the literature, for example given between Mengiardi and Pessis [12, 26].

ROC analysis revealed a significantly higher diagnostic performance of both CE parameters compared to all individual non-enhanced parameters, in line with the findings of Pessis et al. [12] who reported significantly higher sensitivity and specificity of axillary recess CE (sensitivity 97.6%; specificity 97.6%) than for T2-weighted hyperintensity (sensitivity 90.5%; specificity 92.7%). These results are further confirmed by a logistic regression model showing that both CE parameters contributed significantly better than all non-enhanced parameters together. This supports previous findings showing that additional evaluation of contrast-enhanced sequences has a benefit on overall sensitivity and specificity in diagnosis of ACS, especially when considering different levels of radiologist experience [5]. Furthermore, in our current study, each of the CE imaging signs could detect almost all cases (78.6–100%) of ACS, in which individual non-enhanced signs had been negative. Another relevant fact is that CE imaging signs showed highest accuracy when all degrees of enhancement (mild, moderate, and strong) were rated positive.

However, evaluation of individual imaging signs—as described earlier [11, 12]—may only in part reflect clinical practice when radiologists have to decide whether a patient’s MRI is in total indicative for ACS or not. Chi et al. [13] already analyzed a combination of imaging signs, however with some limitations. In their study, accuracy only for a combination of CHL thickening, SCF obliteration, and axillary recess thickening was calculated. SCF obliteration was used as a decisive imaging sign; however, our and other studies (Ahn et al. [11]: sensitivity 90–92; specificity 13–19%) showed only low accuracy for SCF obliteration. In their study, statistical analysis apparently was based on McNemar test for comparison of sensitivities and specificities which only represents a part of the total ROC. In our opinion, a comparison of AUC with confidence intervals and DeLong [23] method based on a regression model is more accurate for this setting of individual and combined imaging signs. Furthermore, no explanation was given on how the combinations were used.

The relevance of combined imaging signs is illustrated by calculating accuracy when the presence of one or more positive non-enhanced imaging signs would be sufficient to diagnose ACS; on the other hand, in this scenario, all non-enhanced imaging signs would have to be negative for diagnosis of not having ACS. While calculated results show excellent sensitivity, there is a heavy penalty of poor TPR and specificity, and therefore, this approach cannot be considered diagnostically relevant. In such a scenario, testing pairwise combinations of individual imaging signs without evaluation of the other poorer performing imaging signs can help increase diagnostic performance of non-enhanced imaging as this method increases the sensitivity and at the same time preserves high specificity; however, this effect was not statistically significant in our study. Indeed, combining AR signal intensity with AR thickness and, even more, AR signal intensity with RI thickness distinctly increases diagnostic performance for all readers, however with slightly reduced TPR compared to evaluation of axillary recess signal intensity alone. An explanation why the combination of axillary recess signal intensity and RI thickness and of axillary recess signal intensity and axillary recess thickness performed distinctly better than all individual signs could be that adhesive capsulitis does not manifest homogenously in all parts of the capsule. This is supported by results of Pessis et al. [12], who found correlation of contrast enhancement of RI with clinical stage but not of AR, or by Sofka et al. [27] who detected an association of AR thickness and signal intensity with clinical staging (highest values in stage 2) while scarring of rotator interval was generally present in all ACS patients but did not show significant differences between clinical stages. At least, this would explain the lack of sensitivity of individual assessment of non-enhanced imaging signs. Diagnostic benefit of combined evaluation of non-enhanced imaging signs is also evident in agreement among readers as kappa of combined axillary recess signal intensity and RI thickness is higher than of individual non-enhanced imaging signs.

However, diagnostic performance of pairwise combined non-enhanced imaging signs is still distinctly outperformed by both individual CE imaging signs and even more by the combination of the two CE imaging signs as shown in Table 2.

ACS typically shows a four-staged course [1, 28] where the two main clinical symptoms are pain and limited active and passive range of motion; however, their dynamics differ across the stages. Typically, pain is most severe in stages 1 and 2 and decreases in stage 3 and 4. Limited range of motion is reported to be most severe in stages 2 and 3 and normally improves in stage 4. In arthroscopy, the synovial layer of the capsule presents typically erythematous and thickened in pain-associated stages 1 and 2 while in stages 3 and 4, the erythematous aspect gives way to an increasingly contracted capsule [1, 26]. Histologically, stages 1 and 2 are characterized by inflammatory cell infiltration and synovial proliferation while in higher stages, dense collagenous tissue develops within the capsule suggesting that inflammation leads to reactive capsular fibrosis [19, 29]. Based on these findings, it can be assumed that especially stages 1 and 2—and to a lesser degree stage 3—are associated with contrast uptake in MRI, whereas in the late fibrotic stage 4, contrast enhancement will usually be missed. Ahn et al. [20] tried to correlate clinical impairment with various MRI findings. In their study, a negative linear correlation was found between thickness of the joint capsule and external rotation as well as a positive correlation between contrast enhancement in the axillary joint capsule and pain.

One limitation of our study is that we could not obtain arthroscopic or histological correlation of clinical and MR morphological findings as most patients with ACS are treated conservatively. Another potential limitation is that due to the clearly designed clinical standard of reference including the presence of at least four out of five clinical signs of ACS, we have probably included almost exclusively cases with clinically clear diagnoses. However, as previously discussed in the literature [5], this applies for all studies on ACS, as no true “golden standard” other than histology has been defined and is in keeping with a conservative estimate of the diagnostic performance of MRI. Moreover, additional disorders in the shoulder can coexist with adhesive capsulitis. However, our study is focused on and designed for the diagnosis of frozen shoulder. Evaluation of other pathologies is beyond the scope of the study design. Exclusion of synovitis and osteoarthritis may be a limitation of the standard of reference, but necessary because clinical symptoms can be very similar to ACS [1]. Another potential limitation might be that the imaging protocol of this study included non-fat saturated T2w sagittal images. Therefore, an increased signal intensity in the capsule in patients with active capsulitis might be missed in these sequences resulting in decreased sensitivity. However, advantages of the T2-weighted images without fat saturation are a better delineation of the CHL and a facilitated depiction of SCF and its obliteration.

Although administration of contrast agent may have implications in relation to extra costs, longer scanning time, and patients’ convenience, we hold it to be justified in view of our results.

## Conclusion

We conclude that using evaluation of CE imaging signs shows both a better agreement among readers with different levels of expertise and a significantly higher performance for diagnosing ACS as compared to non-enhanced MR imaging signs based on the imaging protocol used in this study.

A combined evaluation of parameters showed a tendency to increase discrimination and hence may help in routine diagnosis, especially when some of the many individual parameters are contradictory. However, the effect on diagnosis of ACS was not statistically significant in our study.

## Data Availability

The data that support the findings of this study are available from the corresponding author, BE, upon reasonable request.
